# Effect of roasting conditions on color development and Fourier transform infrared spectroscopy (FTIR-ATR) analysis of Malaysian-grown tropical almond nuts (*Terminalia catappa* L.)

**DOI:** 10.1186/s13065-014-0055-2

**Published:** 2014-09-07

**Authors:** Siewsee Ng, Ola Lasekan, Kharidah Muhammad, Rabiha Sulaiman, Norhayati Hussain

**Affiliations:** Department of Food Technology, Faculty of Food Science and Technology, University Putra Malaysia, UPM, 43400 Serdang, Malaysia; Department of Food Science, Faculty of Food Science and Technology, University Putra Malaysia, UPM, 43400 Serdang, Malaysia

**Keywords:** *Terminalia catappa*, Roasting, Response surface methodology, Scanning electron microscopy, Fourier transform infrared spectroscopy

## Abstract

**Background:**

Proper roasting is crucial to flavor, color, and texture development in the final product. In recent years, several research studies have been carried out to establish the best optimum roasting conditions for some common edible nuts such as; hazelnut, peanut, and pistachio nut. Although roasting is an important process for nuts and oilseeds, there is little or no information on the development of color, aroma, and textural changes in *Terminalia catappa* nuts during roasting.

**Results:**

Results showed that color formation and browning index were significantly (P < 0.05) influenced by the roasting temperature and time of roasting. However, the fracturability of nuts was significantly (P < 0.05) affected by both temperature of roasting and time as well as pH. The optimized results showed that the best response was reached when the roasting time was 29.9 min, roasting temperature 174.5°C, and pH 6.08, respectively. Moreover, the 3400–1560 cm^−1^ carbonyl region for carboxylic acid, alkenes, esters, and amines was found to provide a flavor-print of the roasted tropical almond nut. While, increase in temperature did not produce new carbonyl compounds, it however led to higher concentration of compounds. Scanning electron microscopy of the almond nuts showed that the starch granules were embedded in tissues.

**Conclusion:**

These results showed that roasting temperature and time of roasting were the main variables that significantly affected the physicochemical properties of roasted tropical almond nuts. Moreover the flavor-prints for the roasted nut were produced in the 3400–1560 cm^−1^ carbonyl region.

**Graphical Abstract:**
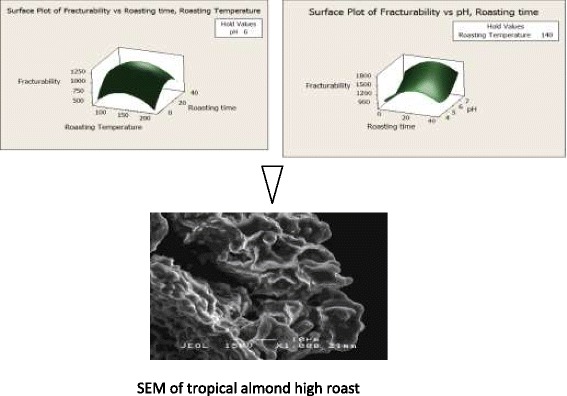
Effect of roasting conditions on fracturability and structural morphology of tropical almond nuts (*T. catappa).*

## Background

On a global basis, almonds ranked number two after cashew nuts in tree nut production with 2,560,000 metric tons in 2010 [1]. According to FAO [2] report, the global consumption of the edible nut was reported as 2.1 kg per person per production. *Terminalia catappa* Linn is a tropical almond nut and a member of the Combretaceae family [3]. The dried nuts are sometimes consumed naturally or in most cases they can be thermally processed prior to consumption.

Hot air roasting is a common practice to which nuts are subjected to before being used as a snack item or before being incorporated into food [4,5]. Roasting is one of the methods used in developing sensorial properties of nuts. It also deactivates enzymes that accelerates nutrient destructions and eliminates unwanted microorganisms, and food contaminants [6–8].

For roasted products, brown color plays an important role in consumer’s acceptability and preferences. In recent years, there have been limited literatures on the kinetic studies of color development in roasted products. Therefore, establishing an optimum brown color for the roasted products is a major objective of roasting [9,10]. Color development has been shown to dependent on factors such as; raw sample’s pH, and the roasting conditions (i.e. roasting temperature and roasting time) [9]. For example, studies have revealed that the optimum roasting conditions for macadamia nuts and peanuts were 135°C for 20 min; and 180°C for 45 min [11,12], respectively.

Besides brown color, texture and aroma are also some of the major characteristics that contribute to the quality of roasted products. The development of brown color and aroma are phenomena that results from the Maillard reaction. Generally, analyses of aroma compounds in roasted nuts have been carried out qualitatively and quantitatively by using gas chromatography–mass spectrometry (GC-MS), and gas chromatography-olfactometry (GC-O) [11–16]. These approaches are time-consuming. Recently, the Fourier Transform Infrared (FTIR)-attenuated total reflection (ATR) spectroscopy has been employed. This is a simple, rapid, high sensitivity and easy to monitor technique. This technique has been used for discriminating different genotypes and origins of roasted coffee, and the degree of roasting temperature [17], cashew nut shell [18], pistachio-nut shell [19], brewed coffee [20] and almond oil [21] respectively.

Proper roasting is crucial to flavor, color, and texture development in the final product. In recent years, several research studies have been carried out to establish the best optimum roasting conditions for some common edible nuts such as; hazelnut [22], peanut [23], and pistachio nut [10]. Although roasting is an important process for nuts and oilseeds, however, there is little or no information on the development of color, aroma, and textural changes in *Terminalia catappa* nuts during roasting. Therefore, this research study is aimed at characterizing the roasting conditions that would produce almond nuts with desirable color, browning index, and fracturability; meanwhile, changes in the functional groups and flavor-print of nuts during roasting were evaluated using Fourier transform infrared (FTIR-ATR) analysis.

## Results and discussion

Results of the response surface analysis showed that the predicted regression coefficients of the fitted mathematical models with the corresponding *R*^*2*^ values, and lack of fit test were between 0.3068 and 0.7806. The significance of the regression models of each response variables was indicated by the confidence level or the commonly called *p*-value. The confidence level of mathematical models that is less than 5% (*p* < 0.05) is considered significant to that particular response variable. The final reduce models were significantly (*p* < 0.05) fitted for the four response variables studied: namely color L, and b, browning index, and fracturability with *R*^*2*^, ranging from 0.4692 to 0.7806. However, the model was insignificantly (*p* > 0.05) fitted for the color ‘a’ which has an *R*^*2*^ = 0.3068 (Table 1). Besides, the ‘goodness of fit’ was evaluated using ANOVA so that only significant (*p* < 0.05) terms were incorporated in the final reduced model (Table 2).

**Table 1:** **Regression coefficients and ANOVA for color (L, a, b), browning index and fracturability**

**Regression coefficient**	**Responses**				
	**Colour**			**Browning index**	**Fracturability**
	**L**	**a**	**b**		
*b*_*0*_	−8.49938	11.9704	6.35601	38.1035	3181.45
*b*_*1*_	0.71584	−0.0714	0.02791	−0.3362	19.47
*b*_*2*_	0.89370	−0.8000	0.04250	-	36.19
*b*_*3*_	-	-	−0.57805	-	−1311.91
*b*_*1*_^*2*^	−0.00212	0.0003	-	0.0015	−0.07
*b*_*2*_^*2*^	-	-	-	-	−0.90
*b*_*3*_^*2*^	-	-	-	-	119.35
*b*_*12*_	−0.00645	-	-	-	-
*b*_*13*_	-	-	-	-	-
*b*_*23*_	-	-	-	-	-
*R* ^*2*^	0.7469	0.8699	0.7909	0.5214	0.7806
*R* ^*2*^ (adj)	0.6794	0.8455	0.7517	0.4651	0.6211
Regression (*P* value)	0.000*	0.000*	0.000*	0.002*	0.008*
Lack of fit (*F* value)	5.93	5.28	7.30	0.23	10.93
Lack of fit (*P* value)	0.009*	0.010*	0.020*	0.796**	0.037*

*b*_*0*_: The estimated regression coefficient for the main linear effect.

*b*_*1*_: The estimated regression coefficient for the quadratic effect.

*b*_*2*_: The estimated regression coefficient for the interaction effect.

(1): Temperature (2): Time (3): pH.

*Significant (*p* < 0.05).

**Not significant (*p* > 0.05).

**Table 2:** **ANOVA and regression coefficients of the first- and second-order polynomial models**

**Variables**	**Main effects**			**Quadratic effects**			**Interaction effects**		
	***X***_***1***_	***X***_***2***_	***X***_***3***_	***X***_***1***_^***2***^	***X***_***2***_^***2***^	***X***_***3***_^***2***^	***X***_***1***_ ***X***_***2***_	***X***_***1***_ ***X***_***3***_	***X***_***2***_ ***X***_***3***_
Colour L									
*P* value	0.000*	0.002*	-	0.000*	-	-	0.001*	-	-
*F* ratio	38.07	14.41	-	28.46	-	-	15.43	-	-
Colour a									
*P* value	0.001*	-	0.000*	0.000*	-	-	-	-	-
*F* ratio	16.35	-	64.15	22.38	-	-	-	-	-
Colour b									
*P* value	0.000*	0.008*	0.005*	-	-	-	-	-	-
*F* ratio	40.40	9.17	10.86	-	-	-	-	-	-
Browning Index									
*P* value	0.078**	-	-	0.028*	-	-	-	-	-
*F* ratio	3.51	-	-	5.77	-	-	-	-	-
Fracturability									
*P* value	0.036*	0.007*	0.038*	0.030*	0.011*	0.025*	-	-	-
*F* ratio	5.68	10.85	5.56	6.17	9.25	6.67	-	-	-

Values of *P* value and *F* ratio less than 0.050 indicate model terms are significant. Values of *P* value and *F* ratio greater than 0.050 indicate model terms are not significant.

*Significant (*p* < 0.05).

**Not significant (*p* > 0.05).

Coded forms for response variables *X*_*1*_: Roasting temperature; *X*_*2*_: Roasting time; *X*_*3*_: pH.

Eq. 1, 2, 3, 4 and 5 showed the fitted models obtained for predicting the response variables as below:

1 $$ \mathrm{Color},\mathrm{L}:{Y}_1 = -8.499+0.716{X}_1+0.894{X}_2-0.002{X_1}^2-0.006{\mathrm{X}}_1{X}_2 $$

2 $$ \mathrm{Color},\ \mathrm{a}:{Y}_2 = 11.970-0.0714{X}_1-0.800{X}_3+0.0003{X_1}^2 $$

3 $$ \mathrm{Color},\ \mathrm{b}:{Y}_3 = 6.356+0.0279{X}_1+0.0425{X}_2-0.578{X}_3 $$

4 $$ \mathrm{Browning}\ \mathrm{index}:{Y}_4 = 38.104-0.3362{X}_1+0.0015{X_1}^2 $$

5 $$ \mathrm{Fracturability}:{Y}_5=3181.45+19.47{X}_1+36.19{X}_2-1311.91{X}_3-0.07{X_1}^2-0.90{X_2}^2+119.35{X_3}^2 $$

### Effect of different roasting variables on color, browning index and fracturability of almond nuts (*Terminalia catappa*)

#### Colors L, a, and b

**Figure 1:**
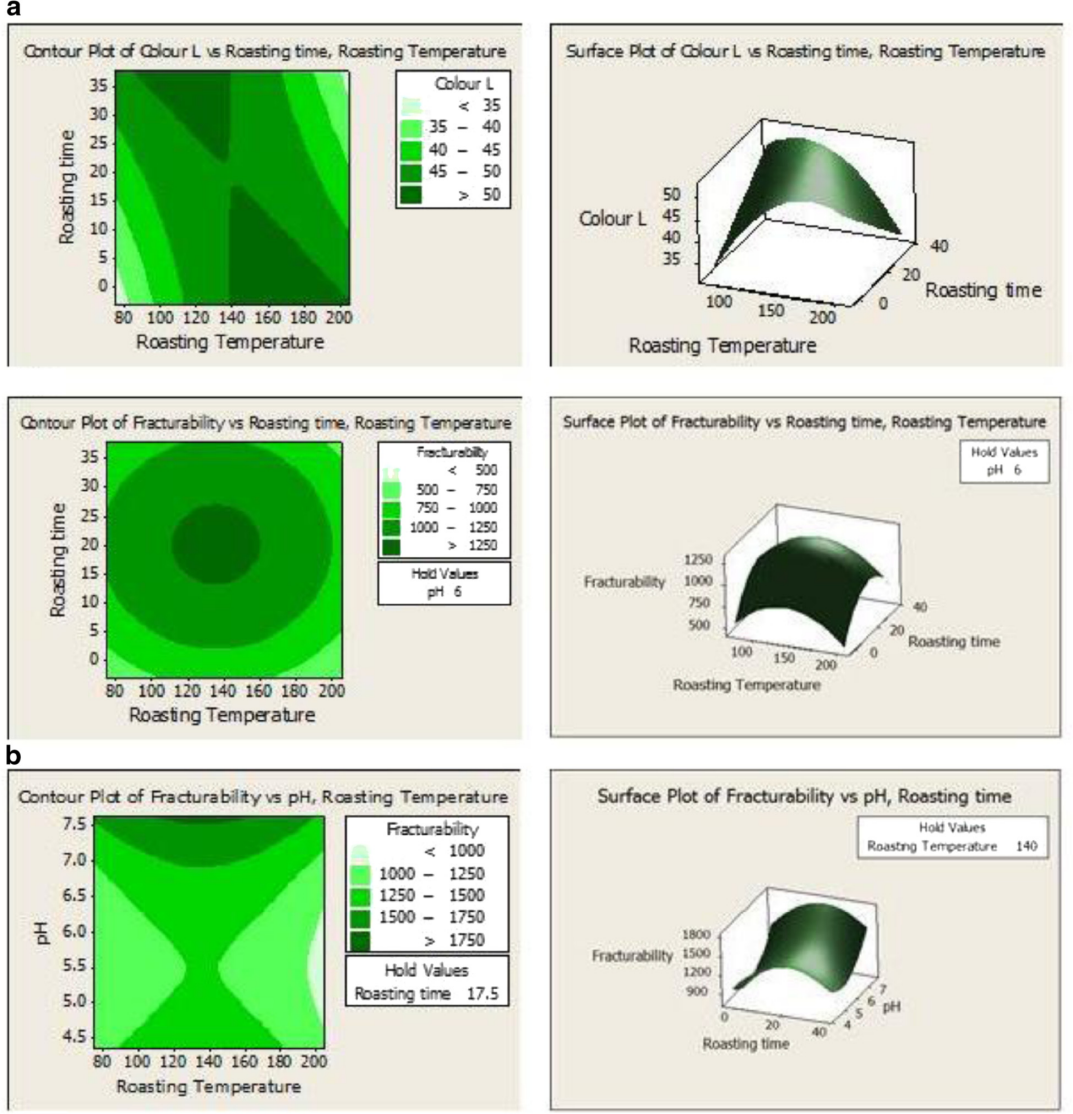
**Effect of roasting conditions (temperature and time) on the (a) Color, L and roasting conditions (temperature, time and pH) on the (b) Fracturability of almond nuts** ***Terminalia catappa.***

As shown in Eq. 1, the second-order polynomial regression Eq. 1 (full quadratic) was used to predict the color L value. As shown in Table 1, the main effect and quadratic terms of roasting temperature significantly affected (*p* < 0.05) color L. Similarly, the color L was also affected by the roasting temperature and time interaction terms. To aid visualization on the effect of roasting condition (roasting temperature and time) on the color L of the almond nuts, the response surface for color L is shown in Figure 1a. The L value revealed an inverse relationship with roasting temperature and time. The L value decreases with roasting which was more pronounced with increasing roasting temperature (Figure 1a). Higher roasting temperatures resulted in a darker color (lower L value) of almond nuts. This browning is a non-enzymatic reaction which occurs when a reducing sugar and protein are heated together [22].

Unlike color L, color a, and b values increased as roasting temperature and time were increased (Table 1). The color changes were most probably not due to enzymatic browning because enzymes associated with enzymatic browning had been destroyed at temperatures ≥100°C. Thus, changes in colors a, and b might be due to internal browning. From Eq. 2 and 3, quadratic term was fitted for predicting the color a value; whereas linear term was fitted for color b. The increase in color a value denotes a redder Chroma, which is indicative of browning reaction [24]. According to Hodge [25], redness of roasted products increases as temperature increases for all yellow materials and this color changes might be associated with Maillard reactions.

Apart from the influence of roasting temperature and roasting time, colors L and b were not significantly (*p* > 0.05) influenced by the pH of the almond nuts; however, pH had significant influence on the color a. Higher pH produces a darker color (i.e. decrease in color L) [26].

### Browning index

The extension of brown color is recognized as browning index (BI) of the products [27]. As observed in Eq. (4), a squared term was fitted for predicting the BI value. Results revealed that the BI value was increased as roasting temperature increased. BI value was insignificantly (*p* > 0.05) influenced by linear effect of roasting temperature; however it was significantly (*p* < 0.05) affected by the squared terms of roasting temperature (Table 1). During roasting, chemical reactions of phospholipid compounds of the nuts enhance the development of brown pigments which give the roasted products darker color, and therefore BI value increased [28]. The optimum BI (25.85) was predicted to be obtained when roasting time was 29.9 min, the roasting temperature was 174.5°C and pH 6.08 respectively.

### Fracturability

As revealed in Table 1, texture (fracturability) of roasted almond nuts was significantly (*p* < 0.05) affected by both the linear and quadratic terms of roasting temperature, time and pH. A surface plot displays the effect of roasting temperature, roasting time and pH on the fracturability (Figure 1b). As observed in Eq. (5), a full quadratic term was fitted for predicting the fracturability value. The 3D surface plot revealed that increasing roasting temperature up to 150°C resulted in increase in the fracturability. However, further increase in temperature led to a gradual decrease in fracturability. Similar trend was observed with time of roasting. Increase in the pH led to significant increases in fracturability. The optimum fracturability (1107.62 g/s) was predicted to be obtained when roasting time was 29.9 min; the roasting temperature was 174.5°C and pH 6.08 respectively.

### Optimization and validation procedures

Both multiple graphical and numerical optimization were established to determine the exact optimum point of the different roasting conditions on the color, browning index, and fracturability of almond nuts leading to the desirable response goals. The final reduced models were expressed as three-dimensional (3D) response surface plot to obtain a better visualization and understanding of the interaction effect of main roasting conditions on the color, browning index and fracturability of almond nuts. The optimum roasting process performed at 29.9 min, 174.5°C and pH 6.08 were recommended for producing roasted almond nuts with optimum quality. The predicted response values for color L, color a, color b, browning index and fracturability were found to be 44.93, 3.64, 8.98, 25.85 and 1107.62, respectively. All response models were verified theoretically. The experimental data were compared with fitted values to verify adequacy of final reduced models by using 2-sample-t-test (results not shown). There was insignificant differences (*p* > 0.05) between experimental and predicted values. This indicates that the predicted models are able to describe the response variables satisfactorily.

### FTIR

**Figure 2:**
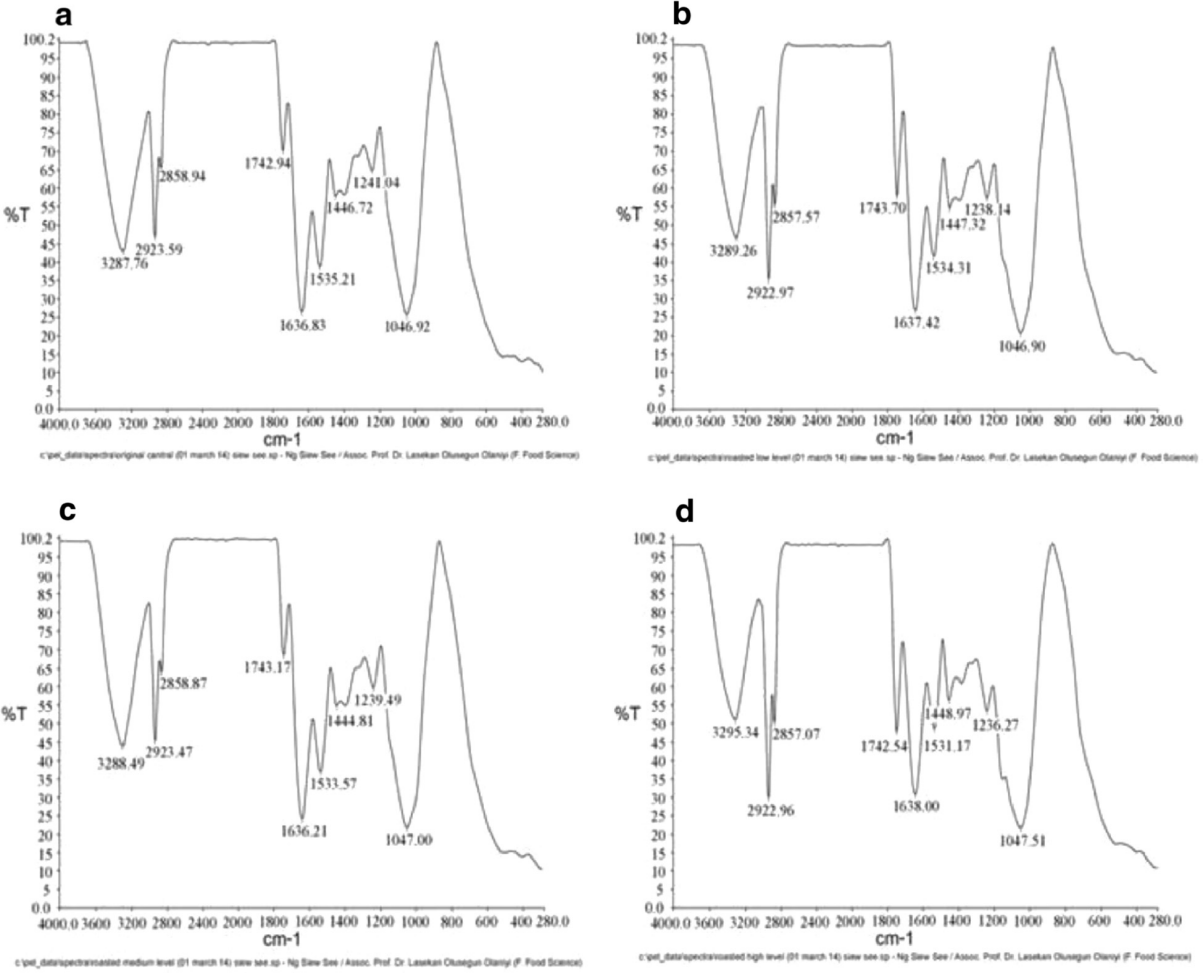
**Fourier transform infrared spectra of raw and roasted** ***Terminalia catappa*** **almond nuts (a) original without roasting; (b) roasted at low level; (c) medium level; (d) high level.**

FTIR spectroscopy-attenuated total reflectance (ATR) technique is useful in converting the emittance spectra to absorbance spectra rapidly, with high-energy throughput without loss of resolution. The FTIR spectra of almond nuts are presented in Figure 2(a) to 2(d) where Figure 2(a) represented spectrum of raw almond nuts without any processing or treatment; Figure 2(b) represented spectrum of roasted almond nuts at low level (100°C for 5 min); Figure 2(c) represented spectrum of roasted almond nuts at medium level (140°C for 17.5 min); and Figure 2(d) represented spectrum of roasted almond nuts at high level (180°C for 30 min).

Figure 2 revealed that both the raw and processed almond nuts’ spectra have the similar band positions which mean that identical compounds were observed in each roast. Thus, the differences among the raw, light, medium and dark roasts were the percentage of transmittance of identical carbonyl compounds instead of the types of carbonyl compounds. The main bands were labeled in the figure and detailed spectral band assignments are given in Table 3.

**Table 3:** **FTIR functional group composition**

**Frequency range (cm** ^**−1**^ **)**	**Frequency (cm** ^**−1**^ **)**				**Group**	**Class of compound**
	**Raw**	**Low level**	**Medium level**	**High level**		
3400-2400	3287.76	3289.26	3288.49	3295.34	O-H stretching	Carboxylic acid
3000-2850	2923.59	2922.97	2923.47	2922.96	C-H stretching	Alkanes
3000-2850	2858.94	2857.57	2858.87	2857.07	C-H stretching	Alkanes
1750-1730	1742.94	1743.70	1743.17	1742.54	C = O stretching	Ester
1640-1560	1636.83	1637.42	1636.21	1638.00	N-H bending	Primary Amines
1550-1450	1535.21	1534.31	1533.57	1531.17	N-H bending	Secondary Amines
1500-1440	1446.72	1447.32	1444.81	1448.97	C-H bending	Alkanes
1300-1000	1241.04	1238.14	1239.49	1236.27	C-O stretching	Ester
1300-1000	1046.92	1046.90	1047.00	1047.51	C-O stretching	Ester

The presence of carboxylic acid existing in almond nuts are indicated by the broad absorbance peak of O-H stretching vibration between 3400 and 2400 cm^−1^. This observation certainly indicates that the compound is a carboxylic acid because the O-H stretch appeared in the spectrum as a very broad band which centers on 3000 cm^−1^ and partially obscures the C-H stretching bands [29]. The presence of alkanes is indicated by the strong absorbance peak of C-H vibrations between 3000 and 2800 cm^−1^ and the C-H deformation vibrations between 1475 and 1350 cm^−1^. Absorption bands at 2922 cm^−1^ and 2857 cm^−1^ correspond to asymmetric and symmetric stretching vibrations of methyl (CH_3_) groups, respectively. The sharp and narrow band observed at 1743 cm^−1^ is assigned to C = O stretching vibration of ester groups in triacylglycerol [30–32].

The absorbance peaks around 1743 cm^−1^ represent the C = O stretching vibration indicating the presence of ester. Although some ester carbonyl groups may appear in the same general area as ketones, ketones can be eliminated in this study by observing the strong and broad C-O stretching vibrations that appeared in region 1300–1000 cm^−1^ where ketonic absorptions appeared as weaker and narrower bands. Two bands appeared for the C-O stretching vibrations in esters in the range from1300-1000 cm^−1^ (Table 3). The aliphatic secondary amines absorbed near 1500 cm^−1^ (about 1535 cm^−1^), the N-H bending vibrations was very weak and usually not observed.

The major differences in the percentage of transmittance (%T) from light roast to medium roast can be found from three compounds: ester, carboxylic acid and amines. The increase in %T was observed in ester (at 1000–1300 cm^−1^); while decreases were observed in the carboxylic acid (around 3289 cm^−1^), ester (around 1743 cm^−1^) and primary and secondary amines (around 1637 and 1535 cm^−1^, respectively). The development of the ester and acid flavor compounds seems to give medium-roasted nuts a more desirable, enhanced and stronger nutty-roasted aroma and flavor compared to light-roasted nuts. Similar results were obtained by Donald *et al.* [20] on the analysis of brewed coffee. The increase in %T in ester might be due to the release of volatiles during roasting of nuts whereas the decrease of %T in primary and secondary amines might be due to the Maillard reaction and the formation of color and aroma. Results of the ‘medium roast’ to ‘dark roast’ revealed major alterations in the transmittance of the carbonyl compounds. There were increases in the %T of carboxylic acid (at 2400–3400 cm^−1^), esters (around 1047 cm^−1^), primary amines (around 1636 cm^−1^) and secondary amines (around 1533 cm^−1^) respectively. There were also decreases in the amount of esters (around 1743 cm^−1^ and at 1236–1240 cm^−1^). These changes are compatible with the sensory panelists’ evaluations of a stronger aroma, taste and aftertaste of nuts. For dark roast, the longer heating time promotes caramelization of sugar. Overall, there was significant increase in %T from raw nuts to roasted nuts in terms of the carboxylic acid, primary amines and secondary amines; however, there was significant decrease in %T for esters formation from raw nuts to roasted nuts.

### Scanning electron microscopy (SEM)

**Figure 3:**
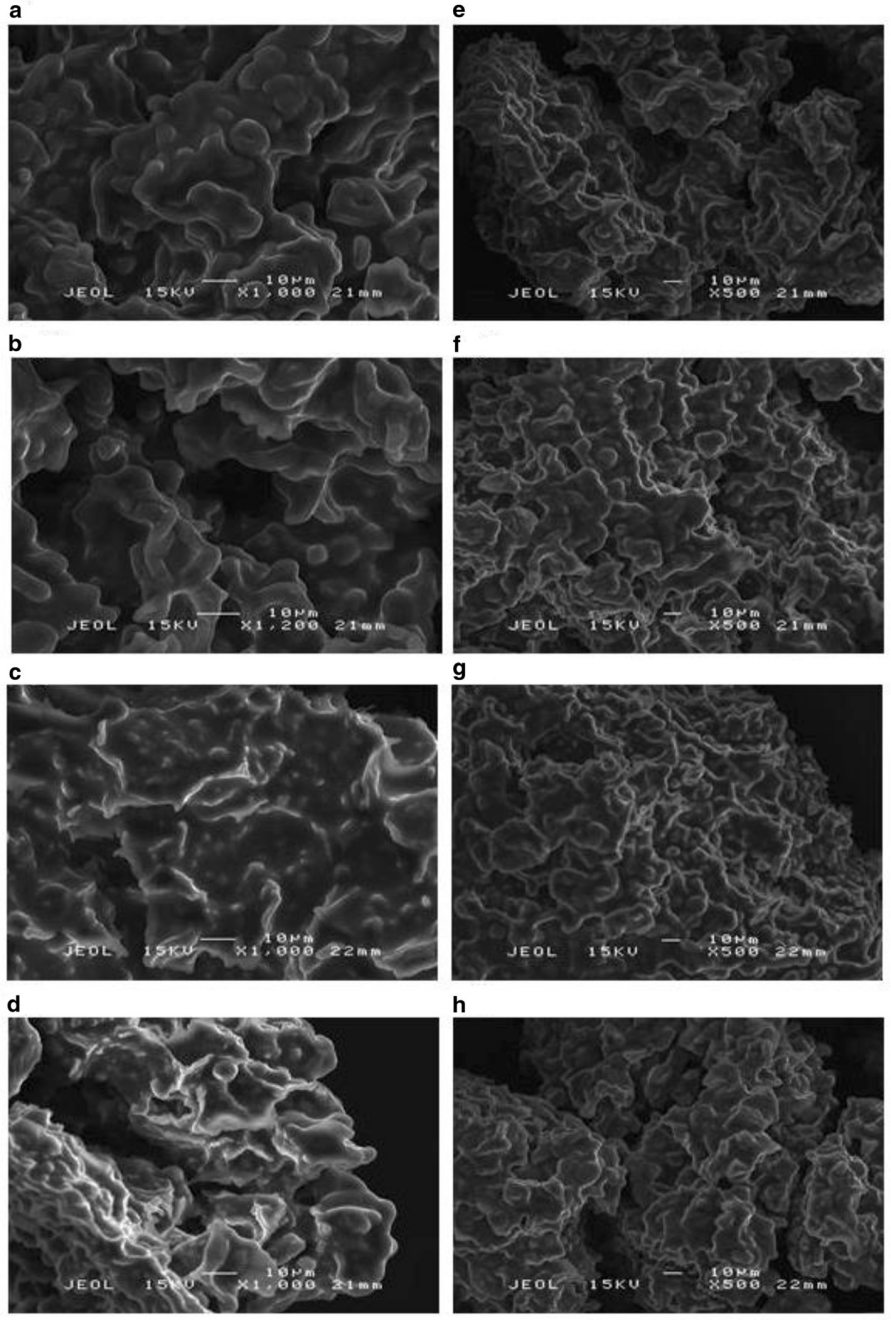
**Scanning electron micrographs at a magnification of x1000 (a)-(d) and x500 (e)-(h), where (a) and (e): raw almond nuts without any heat treatment; (b) and (f): almond nuts roasted at 100°C for 5 min (low level); (c) and (g): almond nuts roasted at 140°C for 17.5 min (medium level); (d) and (h): almond nuts roasted at 180°C for 30 min (high level).**

Scanning electron microscopy (SEM) was used to visualize and monitor the fractural pattern and structural morphology of almond nuts during different stages of roasting process. Few studies have been done using SEM on almond cotyledon [33] and almond protein bodies [34]. The microphotographs of the raw and roasted almond nuts at different heating levels are shown in Figure 3(a)-(h).

The observations revealed that the almond nut is mainly composed of globular structures, where oval-shaped starch granules were distributed throughout the cell. However, this distribution could only be visualized in raw nuts and low-level roasted nuts (100°C for 5 min). The globular structures were possibly starch granules because the starch granules were larger than protein bodies recorded by previous studies [35–37]. Theoretically, protein bodies should be observed from the images captured since nuts are rich in protein (~17%) (Ng, Lasekan, Muhammad, Sulaiman, Hussain: Physicochemical properties of Malaysian-grown almond nuts; forthcoming). However, SEM had limitation in the capability to assess the differences.

For the raw almond nuts (Figure 3(a)), the surface was quite smooth, without any pores, except for some occasional cracks. Heating treatment on the almond nuts from low temperature (100°C) to high temperature (180°C) changed the smooth surface into rougher surfaces (Figure 3b-d). Without roasting, the almond nuts contained moisture content that maintained and supported the structure of the granules, thus the surface was smooth and there were more granules in large sizes compared to those roasted at high temperatures. As roasting temperature increased, the moisture was evaporated and diffused out from the sample. This process caused the larger globules to disintegrate and micropores seem to be developed on the surface indicating the release of volatile matter. In terms of compactability, the granules became more compact after roasting from 100°C to 140°C as shown from Figure 3(e) to (h).

### Sensory evaluation

**Figure 4:**
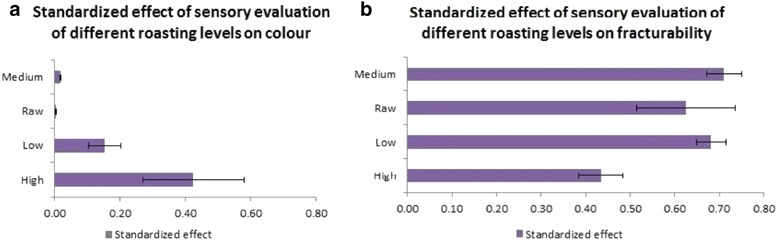
**Pareto chart for responses a: color and b: fracturability of almond nuts at different roasting levels (low, medium, high, and without heat treatment-raw).**

Figure 4 showed the standardized effect of sensory evaluation of differently roasted nuts (i.e. low, medium and high roast) on color and fracturability. Figure 4(a) indicated that most of the panelists preferred the color of almond nuts roasted at high level (180°C for 30 minutes); while Figure 4(b) indicated that most panelists preferred the fracturability of almond roasted at medium level (140°C for 17.5 minutes). From the standardized effect, the optimum roasting level that was preferred by the panelists was in the range of medium and high level.

## Conclusion

The present study showed that the color formation and browning index in roasted tropical almond nuts were significantly (*p* < 0.05) influenced by roasting temperature and roasting time; while the fracturability of roasted almond nuts was significantly (*p* < 0.05) influenced by roasting temperature, roasting time and pH. The optimum roasting process was attained at 29.9 min, 174.5°C, and pH6.08. Moreover, the 3400–1560 cm^−1^ carbonyl region for carboxylic acid, alkenes, esters, and amines was found to provide a flavor-print of the roasted tropical almond nut. Increasing the roasting temperature as observed with the different roasts did not produce new carbonyl compounds, but led to increases in concentration as reflected by the percentage of transmittance of compounds. Scanning electron microscopy of the almond nuts showed that the starch granules were embedded in tissues.

### Experimental

Optimization of roasting conditions (temperature, time and pH) of tropical almond nuts was carried out using RSM [38,39]. A central composite design (CCD) was utilized to study the effects of the three roasting conditions (i.e. pH, roasting temperature and roasting time) on the response variables (brown color, browning index and fracturability), create fitted models among the variables, and optimize the roasting conditions for the optimum production of roasted nuts in terms of desirable brown color, and texture. A three-factor level and a three level face-centered CCD of 20 experimental runs was used (Table 4). The following were the independent variables levels: (1) roasting temperature (100, 140 and 180°C), (2) roasting time (5, 17.5 and 30 min) and (3) pH (5, 6 and 7).

**Table 4:** **The matrix of central composite design (CCD) and experimental data obtained for the response variables studied (** ***Y***_***1***_ **-** ***Y***_***5***_ **) (mean ± SD)**

**Treatment runs**	**Blocks**	**Independent variables**			**Response variables**				
		**Temperature (°C),** ***X***_***1***_	**Time (min),** ***X***_***2***_	**pH,** ***X***_***3***_	**Colour L,** ***Y***_***1***_	**Colour a,** ***Y***_***2***_	**Colour b,** ***Y***_***3***_	**Browning index,** ***Y***_***4***_	**Fracturability (g/s),** ***Y***_***5***_
8	2	100.00	30.00	5.00	53.10 ± 0.54^a^	3.85 ± 0.05^c^	8.41 ± 0.17^b^	22.41 ± 0.23^c^	1315.48 ± 3.36^b^
9	2	100.00	5.00	7.00	43.85 ± 0.08^d^	2.19 ± 0.04^e^	5.32 ± 0.07^e^	16.50 ± 0.22^d^	1221.65 ± 3.23^c^
10	2	180.00	30.00	7.00	47.17 ± 0.45^c^	2.85 ± 0.12^d^	9.04 ± 0.29^a^	25.51 ± 0.71^b^	1546.31 ± 15.09^a^
11(*C*)	2	140.00	17.50	6.00	48.41 ± 0.39^c^	2.82 ± 0.07^d^	7.39 ± 0.07^c^	20.70 ± 0.19^c^	1289.27 ± 5.87^c^
12(*C*)	2	140.00	17.50	6.00	49.12 ± 0.29^bc^	2.83 ± 0.13^d^	7.23 ± 0.11^c^	19.80 ± 0.06^d^	1419.53 ± 20.03^b^
7	2	180.00	5.00	5.00	48.67 ± 0.08^c^	4.79 ± 0.09^a^	8.51 ± 0.08^b^	26.12 ± 0.50^b^	1117.77 ± 8.20^d^
2	1	180.00	30.00	5.00	41.71 ± 0.09^d^	4.97 ± 0.09a	9.76 ± 0.27^a^	35.18 ± 1.10^a^	920.68 ± 17.92^e^
6(*C*)	1	140.00	17.50	6.00	49.40 ± 0.32^bc^	2.79 ± 0.08^d^	7.33 ± 0.21^c^	20.06 ± 0.49^c^	1247.72 ± 11.96^c^
5(*C*)	1	140.00	17.50	6.00	50.78 ± 0.42^b^	3.00 ± 0.06^d^	7.98 ± 0.08^c^	21.28 ± 0.11^c^	1216.48 ± 14.16^c^
4	1	100.00	30.00	7.00	51.21 ± 0.41^b^	2.22 ± 0.02^e^	6.79 ± 0.15^d^	17.29 ± 0.24^d^	1147.99 ± 17.68^d^
3	1	180.00	5.00	7.00	49.37 ± 0.57^bc^	2.82 ± 0.05^d^	6.59 ± 0.11^d^	18.40 ± 0.20^d^	1430.61 ± 17.06^b^
1	1	100.00	5.00	5.00	43.82 ± 0.06^d^	4.41 ± 0.07^b^	7.02 ± 0.04^d^	24.68 ± 0.08^b^	1146.64 ± 8.53^d^
20(*C*)	3	140.00	17.50	6.00	50.54 ± 0.35^b^	3.22 ± 0.03^d^	7.52 ± 0.09^c^	20.65 ± 0.02^c^	1251.09 ± 16.27^c^
13	3	74.68	17.50	6.00	36.75 ± 6.37^e^	3.11 ± 0.14^d^	4.84 ± 0.15^e^	20.21 ± 0.56^c^	981.28 ± 19.17^e^
19(*C*)	3	140.00	17.50	6.00	49.10 ± 0.32^bc^	2.93 ± 0.06^d^	7.27 ± 0.04^c^	20.27 ± 0.25^c^	1302.49 ± 16.28^b^
17	3	140.00	17.50	4.37	51.76 ± 1.03^b^	4.18 ± 0.08^b^	8.49 ± 0.31^b^	23.69 ± 0.24^b^	1215.75 ± 16.54^c^
16	3	140.00	37.91	6.00	45.53 ± 0.49^d^	2.61 ± 0.03^de^	7.06 ± 0.21^d^	20.91 ± 0.33^c^	991.15 ± 5.14^e^
18	3	140.00	17.50	7.63	50.49 ± 0.54^b^	1.51 ± 0.03^f^	7.42 ± 0.12^c^	24.86 ± 0.12^b^	1679.96 ± 23.81^a^
15	3	140.00	−2.91	6.00	51.06 ± 0.37^b^	2.37 ± 0.11^e^	6.74 ± 0.25^d^	17.46 ± 0.84^d^	518.60 ± 10.11^f^
14	3	205.32	17.50	6.00	42.88 ± 1.29^d^	5.11 ± 0.18^a^	10.06 ± 0.44^a^	34.93 ± 0.93^a^	665.62 ± 12.26^f^

(*C*): Centre point.

Coded forms for response variables *X*_*1*_: Roasting temperature; *X*_*2*_: Roasting time; *X*_*3*_: pH.

^a-f^: Means with different superscripts along the same column are significantly different (P <0.05).

## Materials

The tropical almond fruits were collected between January and February, 2014 from the forestry Department of the University Putra Malaysia, Serdang. In all, about two thousands almond seeds were obtained.

### Chemicals

37% Hydrochloric acid, 0.01 N NaOH were purchased from Merck.

### Sample and preparation

The nuts were carefully cracked after *Terminalia catappa* fruits were dried under the sun for two weeks. The dried samples were then milled using a blender to produce powder prior to use. Almond nuts at pH5-7 (approximately 100 g for each treatment) were roasted at 100, 140 and 180°C for 5, 17.5 and 30 min in an oven (Elba) (Table 5). Roasted samples were allowed to cool to room temperature (29 ± 2°C) before analysis.

**Table 5:** **Independent variables and levels established through the central composite design for nuts roasting conditions**

**Independent variables**	**Independent variables level**				
	**Low**	**Medium**	**High**	**Axial (−** ***α*** **)**	**Axial (** ***+α*** **)**
Roasting temperature (°C)	100.0	140.0	180.0	74.68	205.32
Roasting time (min)	5.0	17.5	30.0	−2.91	37.91
pH	5.0	6.0	7.0	4.367	7.633

Axial points allow for the estimation of the tuning parameters of a second-order model. CCD is afirst-order (2^N^) designs augmented by additional centre and axial points.

### Color and browning index measurement

The L, a, and b values are the three dimensions of color which gives specific values of the sample tested [40]. Color L value represents light–dark spectrum with a range from 0 (black) to 100 (white); color a value represents greenness to redness spectrum with the range from −60 (green) to +60 (red); while color b value represents blueness to yellowness spectrum with the range from −60 (blue) to +60 (yellow). From the readings of L-, a- and b- values, the browning index (BI) was calculated using Equation 6 [27].

6 $$ \begin{array}{l} BI = \left[100\left(x-0.31\right)\right]/0.17\\ {}\mathrm{Where},x = \left(\mathrm{a}+1.75L\right)/\left(5.64L+\mathrm{a}-3.012b\right)\end{array} $$

### Texture profile analysis

Fracturability of the roasted almond nuts was analyzed using a Universal Texture Analyzer (CNS, Farnell, UK) equipped with the Texture Pro™ texture analysis software. A 2 mm diameter cylinder probe P/2, with a 20 mm height was used for the measurement of texture. The probe was allowed to penetrate about 3 mm through the sample at 1 mm/s with trigger 5 g. The texture profile analyzer was able to provide the fracturability readings of the sample nuts. Fracturability (g/s) (i.e. first peak of first compression) was used to evaluate the textural properties of the almond nuts. Three replications were performed (i.e. three almonds from each of the roasting levels).

### Statistical analyses

Multiple regression analysis and analysis of variance (ANOVA) were carried out to determine the most fitted response surface models by using the obtained data from the experiment to analyze the optimum region for the response variables. The main and quadratic polynomial models for the response variables were predicted using the least-squared method [41]. The regression polynomial equation (Eq. 7) was used to evaluate the behavior of the response surface for the response function *Y*_*1*_, and the predicted response respectively.

7 $$ \begin{array}{l}{Y}_1={\beta}_0+{\beta}_1{x}_1+{\beta}_2{x}_2+{\beta}_3{x}_3+{\beta}_{1 1}{x}_{1 2}+{\beta}_{2 2}{x}_{2 2}+{\beta}_{3 3}{x}_{3 2}\\ {}+{\beta}_{1 2}{x}_1{x}_2+{\beta}_{1 3}{x}_1{x}_3+{\beta}_{2 3}{x}_2{x}_3\end{array} $$

### Verification of models

A comparison between the experimental and fitted data predicted by the response regression models was used to check the adequacy of the response surface equation. The experimental response data were shown to be in agreement with the predicted. Closeness with the predicted and experimental data confirmed the adequacy of the corresponding response surface models used to describe the variations of response variables as functions of roasting conditions.

### Scanning electron microscopy (SEM)

The shape and surface morphology of raw, and three differently roasted almond nuts samples, were examined by scanning electronic microscopy (SEM). A small amount of dried powders were spread on aluminum stubs. The stub containing the sample was placed in the SEM chamber and coated with palladium with an auto-fine coater for 180 seconds; specimens were viewed with a JEOL JSM 6400 SEM Attached to EDX (Energy Dispersive X-ray) at working distance of 22 mm and an accelerating voltage of 15 kV.

### Fourier transform infrared (FTIR) spectroscopy

The IR spectra were collected using PerkinElmer Spectrum 100 Series spectrometer (United Kingdom) facilitated with a mid-infrared detector- DTGS (deuterated triglycine sulphate). Surface functional groups of the nuts were detected using FTIR. The samples were dispersed in potassium bromide pellet and compressed into discs by pressure. Then, the samples were placed in the light path to allow the infrared light to pass through them and the spectrum was obtained. The spectra were recorded in the region of 4,000 to 280 cm^−1^, with a resolution of 4 cm^−1^.

### Sensory evaluation

Five attributes (aroma, color, fracturability, flavor and overall acceptability) of the almond nuts samples were evaluated by 18 trained panelists from the University Putra Malaysia. Four freshly prepared almond nuts (i.e. dried nuts, and three differently roasted nuts) were presented in air-tight containers coded with three-digit numbers, covered and presented to each panelist. Panelists assigned scores to samples using a nine-point hedonic scale for all the attributes (1 = like extremely, 9 = dislike extremely). Hierarchical multiple regression and Pareto charts were established to analyze the outcomes of the sensory evaluation.
